# Oral and Cutaneous Melanoma: Similarities and Differences

**DOI:** 10.4021/jocmr416w

**Published:** 2010-08-18

**Authors:** Rafaela Nogueira Moreira, Cassio Roberto Rocha Santos, Nadia Lages Lima, Flaviana Dornela Verli, Sandra Aparecida Marinho

**Affiliations:** a Postgraduate Program in Dentistry, Federal University of the Jequitinhonha and Mucuri Valleys (UFVJM), Diamantina, Minas Gerais, Brazil; b Postgraduate Program in Dentistry, Pathology Laboratory, Federal University of the Jequitinhonha and Mucuri Valleys (UFVJM), Diamantina, Minas Gerais, Brazil

## Abstract

Melanomas are malignant lesions stemming from the disorganized proliferation of melanocytes. This condition is more common on skin, but may also be detected in mucosa, such as in the oral cavity. The aim of the present study was to report similarities and differences between oral and cutaneous melanoma.

**Keywords:**

Melanoma; Skin; Mouth; Diagnosis

## Introduction

Melanoma originates from the malignant transformation of melanocytes [1]. Oral melanoma is not as common as cutaneous melanoma and has the worst prognosis among malignant tumors [2, 3]. Due to the greater tendency toward metastasis, oral melanoma is considered quite aggressive [4, 5]. Early diagnosis is therefore important [6]. Fortunately, both the oral cavity and skin is of easy access to exams, which may increase the chances of survival [7]. Early diagnosis and treatment are essential for a better prognosis regarding melanomas and reduced risk of mortality [8-10].

As oral melanoma may be asymptomatic in the early stages, it is often only perceived after the appearance of symptoms such as pain, bleeding and ulceration [9]. Most cases of oral melanoma (66.6%) were diagnosed in advanced stages, with lesions greater than 4 cm in diameter; distant metastases were encountered, especially in the lungs; and mean survival was 16.9 months, with only 6.6% of patients surviving more than five years [11].

The aim of the present study was to report the similarities and differences between oral and cutaneous melanoma.

## Review and Discussion

According to Rivers [12] (1996), a single melanoma occurs only occasionally. However, two lesions, with one as a satellite to the main lesion, are relatively common [9]. This occurs due to embolic propagation to the lymph vessels, with the development of secondary tumors a short distance from the primary tumor [13].

Oral melanomas are more commonly found in the maxilla, especially in the palate and gums [1, 4-6, 14-16]. The mandible is only involved in 20% of cases [5, 17]. The preferential location of melanoma in the head and neck is the nasal cavity, which may explain why oral melanoma is more common in the palate, considering the proximity and embryological origin. As these two structures are continually exposed to inhaled air, it is possible that irritants and carcinogenic compounds in the air, such as the components of cigarette smoke, play a contributing role in the development of lesions in these sites [2]. However, the relative role of toxic substances, medications and hormones, such as during pregnancy and the use of hormonal contraceptives, remains unclear. Immune status also plays a determinant role in the course of melanoma, considering its rapid progression in immunosuppressed individuals [18].

Unlike cutaneous melanoma, the pathogenesis and etiogenesis of mucosal melanoma are not yet clear or defined [1, 8, 15, 17, 19]. Sun exposure is not related to the etiology of oral melanoma, but is clearly linked to cutaneous melanoma. Factors such as family history, syndromes, cytogenetic defects, growth factors, pre-existing lesions, mechanical trauma, denture use, infection, oral habits, self-medication, eating disorders, smoking habits and exposure to formaldehyde and other carcinogenic substances may have some etiological significance [5, 20]. Racial, cultural and geographic factors may also predispose individuals to the disease [20]. Japanese, African, American and Hispanic individuals are more commonly affected by oral melanoma, with a greater predilection for the male gender [1, 5, 21, 22]. However, Pour et al [6] (2009) reported a greater prevalence of oral melanoma among women.

The genetic theory regarding the cause of the malignant transformation of pigmented benign tumors is founded on the expression of certain antigens during the transformation process of the benign melanocytic nevus to melanoma, with an alteration in the p53 protein identified in two-thirds of cases. Cytogenetic analysis of a specific gene in melanocytes seems to be very useful to the understanding of the pathogenesis [19]. According to Tanaka et al [23] (2001), the biological behavior of melanoma may be associated to the expression of the proteins Rb, pRb2/p130, p53 and p16, which may be useful in predicting the appearance of this neoplasm.

Melanoma may present as either a flat or nodular, painless, dark brown or black lesion with erythema, ulceration and bleeding [8, 21]. The invasion of the conjunctive tissue with atypical melanocytes alters the configuration of the surface of the lesion. Initially, macular melanoma develops in situ and may later become nodular [24]. Gorsky et al [2] (1998) found that oral lesions were more often diagnosed as nodules, with only 40% of patients complaining of pain or discomfort. In the clinical aspect, the majority of such lesions are described as non-pigmented. Bone erosion is common as the disease progresses [4, 21]. Deep extension, with or without bone invasion, is present in the diagnosis of many cases. However, surface dissemination prior to vertical dissemination is possible and one third of patients with oral melanoma exhibit previous benign melanin pigmentation [25].

An important issue in the management of oral melanoma is to exclude the possibility of its being a metastasis of a cutaneous melanoma, which would affect the determination of the treatment [21]. Greene et al [26] (1953) proposed three useful criteria in the diagnosis of primary oral melanoma: the presence of malignant melanoma in the oral mucosa; the exclusion of melanoma in any other primary site; and the histopathological determination of junction activity, which is described as melanocytes arranged throughout the basal layer of the surface epithelium.

The Clark and Breslow classifications are the most often used assessment systems for the prognosis of cutaneous melanoma [27, 28]. While the Clark classification assesses the depth of the invasion, the Breslow measurement system analyzes the thickness of the tumor of greatest depth to the beginning of the granular layer. These two classification systems have not been validated as prognostic predictors of oral melanoma, likely due to the rarity of this lesion. Moreover, unlike cutaneous melanoma, more oral melanomas are larger than 4 mm upon initial presentation [6].

Prasad et al [29] (2004) established a three-level micro-stage system: Level I - in situ melanoma either with no evidence of invasion or with the presence of individual or agglomerated invasive melanocytes with less than 10 atypical melanocytes near the subethelial junction; Level II - melanoma cells limited to the lamina propria; and Level III - invasion of the deep conjunctive tissue, including skeletal muscle, bone or cartilage. Barker et al [30] (1997) presented a variation of the histological classification: in situ melanoma, restricted to the epithelium; invasive melanoma, extending to the connective tissue; and mixed melanoma, which is a combination of the in situ and invasive forms.

A number of factors may contribute toward a poor prognosis for oral melanoma, including the absence of symptoms in the early stage of the disease and difficulty determining the width of the radial excision due to anatomic limitations and rich blood supply to the region, which may facilitate hematogenic propagation [4, 7, 15, 19, 21, 22, 25]. Lymph nodes, lungs, liver and brain are common sites of metastasis and are often involved in advanced stages of the disease [7, 8].

The five-year survival rate for oral melanoma is generally only between 10% and 25% due to the late detection stemming from the fact that this lesion is less visible than cutaneous melanoma [10]. Lymphatic metastasis at the time of diagnosis seems to be the most important prognostic factor for oral melanoma [8]. Early diagnosis and treatment can reduce the chances of mortality. If diagnosed early enough, when the malignant cells are limited to the epidermis or when there is minimal invasion, melanoma is 100% curable through surgery or is associated to a 95% five-year survival rate for lesions under 1 mm in thickness without ulceration. In contrast, the five-year survival rate for cutaneous melanoma with lesions larger than 4 mm in thickness and ulceration is only 45% [31].

Unlike with cutaneous melanoma, excision biopsy is not feasible for oral melanoma due to the presence of teeth and bone in the region [15]. Aggressive resection with the complete removal of the tumor is also hindered for this reason and incomplete resection contributes toward recurrence or metastasis [4].

As with the cutaneous form, oral melanoma can usually be diagnosed with hematoxylin-eosin staining, which allows easy identification of the disease through its junction activity, diffuse arrangement of spherical or spiny cells with abundant eosinophilic cytoplasm marked by cellular atypia, eosinophilic nucleolus and abundant mitotic figures [32]. However, if the lesion is completely devoid of pigment and the clinical hypothesis of melanoma is not posed, immuno-histochemical analysis using the protein markers S-100, gp-100, HMB-45 and mart-1 is useful [10, 32]. Amelonotic melanoma is rare and has a worse prognosis in comparison to pigmented lesions due to the delayed establishment of the correct diagnosis and onset of treatment [6, 7].

Surgery is the most indicated form of treatment for melanoma. Depending on the stage, chemotherapy and radiotherapy may be used as adjuvant treatment. However, when there is metastasis, melanoma is incurable in most cases [9, 11, 13]. Radiotherapy may be used as a complementary treatment following surgery or when previous treatment has failed [2]. Radiotherapy may also be used as the only treatment in elderly patients or in the preoperative phase for the reduction of the tumor and alleviation of pain symptoms in cases of bone metastasis [13]. Umeda and Shimada [33] (1994) adopted a protocol that consists of surgery of the lesion through oral access, with a 1.5 cm margin of safety, excision of any lymph node with regional metastasis and the administration of chemotherapy. According to Smith et al [13] (1993), a 1cm margin is likely adequate for tumors of the oral cavity and a combination of treatments may provide greater benefits to the patient.

Experimental studies carried out on mice and in vitro have demonstrated that hollow nanoparticles made of gold aggregated to peptides have an affinity for the receptors of melanoma cells and penetrate these cells. Following injection into the organism and an infrared light bath, the cancer cells with nanoparticles in their interior are destroyed by photothermal damage, with the loss of the cell membrane and protein denaturation. This treatment is known as plasmonic photothermal therapy [34]. Gene therapy with the use of modified melanoma cells containing specific genes that code for cytokines, interferon and growth factors may be administered, such as tumor cell vaccines [12]. Immunotherapy may also be incorporated to melanoma treatment, although patient immune response may be significantly altered by the clinical course of the neoplasm [35]. Interleukin-2, interferon alpha, tumor-infiltrating lymphocytes, bacillus Calmette-Guerin, irradiated allogeneic tumor cells and monoclonal antibodies can be used [36].

Preoperative investigations, such as chest x-ray, kidney function test and abdominal ultrasound, should be performed for the identification of metastasis. In the determination of bone metastasis, computed tomography reveals the extent of invasion into structures adjacent to the mouth, such as the nasal cavity or eye, and a possible recurrence of metastasis in regional lymph nodes. When there is distant metastasis due to hematogenic propagation, aggressive surgery is not indicated and either only local palliative excision or no surgery is recommended. Regional metastasis is uncommon, but, when it occurs, there is local recurrence and distant metastasis in the majority of cases [13]. For melanomas greater than 1 mm in thickness, ultrasonography of the lymph nodes and abdomen (including the pelvis and retroperitoneum) are indicated. The first five years following surgery are the most important, with 90% of all cases of metastasis occurring in this period [18].

Health care professionals should be attentive to pigmented lesions in the mucosa as well as skin, carrying out detailed, discerning exams. Mucosal melanoma, especially in the oral cavity, is rare, but is more aggressive and has a poorer prognosis. Nonetheless, if diagnosed early, complete cure is possible.
